# Case report: Successful treatment of a patient presenting with a very rare association of acute lymphoblastic leukemia and mucopolysaccharidosis type IVA

**DOI:** 10.3389/fped.2025.1644720

**Published:** 2025-09-04

**Authors:** Sofia Maria Carlotta Arnaboldi, Martha Caterina Faraguna, Antonella Colombini, Alessandra Sala, Veronica Leoni, Marco Spinelli, Giacomo Gotti, Laura Rachele Bettini, Viola Crescitelli, Anna Commone, Serena Gasperini, Carmelo Rizzari

**Affiliations:** ^1^ Department of Pediatrics, Pediatric Hematology-Oncology Unit, Fondazione IRCCS San Gerardo dei Tintori, Monza, Italy; ^2^ Fondazione Matilde Tettamanti e Menotti De Marchi Onlus, Monza, Italy; ^3^ Department of Pediatrics, Fondazione IRCCS San Gerardo dei Tintori, Monza, Italy; ^4^ School of Medicine and Surgery, University of Milano-Bicocca, Monza, Italy; ^5^ Rare Disease Centre, Fondazione IRCCS San Gerado dei Tintori, Monza, Italy

**Keywords:** acute lymphoblastic leukemia, mucopolysaccaridosis, Morquio a syndrome, B-lineage acute lymphoblastic leukemia, enzyme replacement therapy (ERT), chemo-radiotherapy

## Abstract

Treating Acute Lymphoblastic Leukemia (ALL) in patients with genetic disorders poses significant challenges for onco-hematologists. Mucopolysaccharidosis type IVA (MPS-IVA) is a lysosomal storage disorder that clinically manifests with progressive and multi-systemic comorbidities, primarily affecting the bone, cartilage, spine, heart and lungs. We report a unique case of B-lineage ALL in a patient with MPS-IVA, who was successfully cured with a personalized chemo-radiotherapy approach. The treatment strategy aimed to balance a curative-intent chemotherapy attempt with the minimization of life-threatening complications. This case highlights the importance of individualized therapy in managing ALL in the context of complex comorbidities.

## Introduction

Acute Lymphoblastic Leukemia (ALL) is the most common malignancy in childhood (1). Currently, 5-year overall survival rates for pediatric ALL are reported to exceed 90% across several treatment protocols (1). However, certain subgroups of patients continue to experience inferior outcomes. Among these, children with genetic disorders have usually poorer survival rates compared to other cohorts (2–4). The treatment of ALL in patients with concomitant genetic syndromes is associated with major challenges. In patients with preexisting organ dysfunction, a personalized treatment plan is often required and must be tailored on a case-by-case basis. In this regard, patients with mucopolysaccharidosis type IVA (MPS-IVA) present with a multi-systemic progressive disease, with the bone and cartilage, spine, heart and lungs being the most affected organs (5). MPS-IVA is a lysosomal storage disorder caused by mutations in the *GALNS* gene (NM_000512.5), which leads to a deficiency of the enzyme N-acetyl-galactosamine-6-sulfate sulfatase (GALNS) and the accumulation of glycosaminoglycans (GAG) keratan sulfate and chondroitin 6-sulfate. Phenotypically, MPS-IVA is prominently characterized by short stature and skeletal dysplasia. Further manifestations of the disease consist in restrictive lung disease, hepatomegaly, recurrent airway infections, hypoacusia, corneal clouding, valvular heart disease and dental abnormalities. The central nervous system (CNS) is not directly involved by GAG accumulation and subjects present normal intelligence; nevertheless, patients have a high risk of developing neurological complications due to atlanto-occipital instability and spinal cord compression secondary to extra-dural GAG accumulation, leading to cervical myelopathy and quadriparesis. No curative treatment is available for MPS-IVA. The benefit of hematopoietic stem cell transplantation—the gold standard treatment for mucopolysaccharidosis type I (Hurler syndrome)—is controversial in Morquio syndrome (6). In this context, Enzyme Replacement Therapy (ERT) with elosulfase alfa emerged as a therapeutic option and was approved by EMA and U.S. FDA in 2014. ERT was associated with a decrease in GAG accumulation, stability in motor performance and an improvement in forced vital capacity, but had no effect on skeletal disorders (7). Although mucopolysaccharidoses are not typically associated with an increased risk of malignancy, cancers have been reported in other inherited metabolic storage disorders, including multiple myeloma in Gaucher disease (8) and hepatocellular carcinoma in glycogen storage disease type I (9). Among MPS, only one previous case of Acute Myeloid Leukemia was reported in a 2.5-year-old girl with Hurler syndrome (10). However, that patient died one week after diagnosis. The case reported here represents the first patient with MPS-IVA and B-cell precursor ALL (B-ALL), who was successfully treated with a conventional chemotherapy-based regimen.

## Case presentation

**Figure 1:**
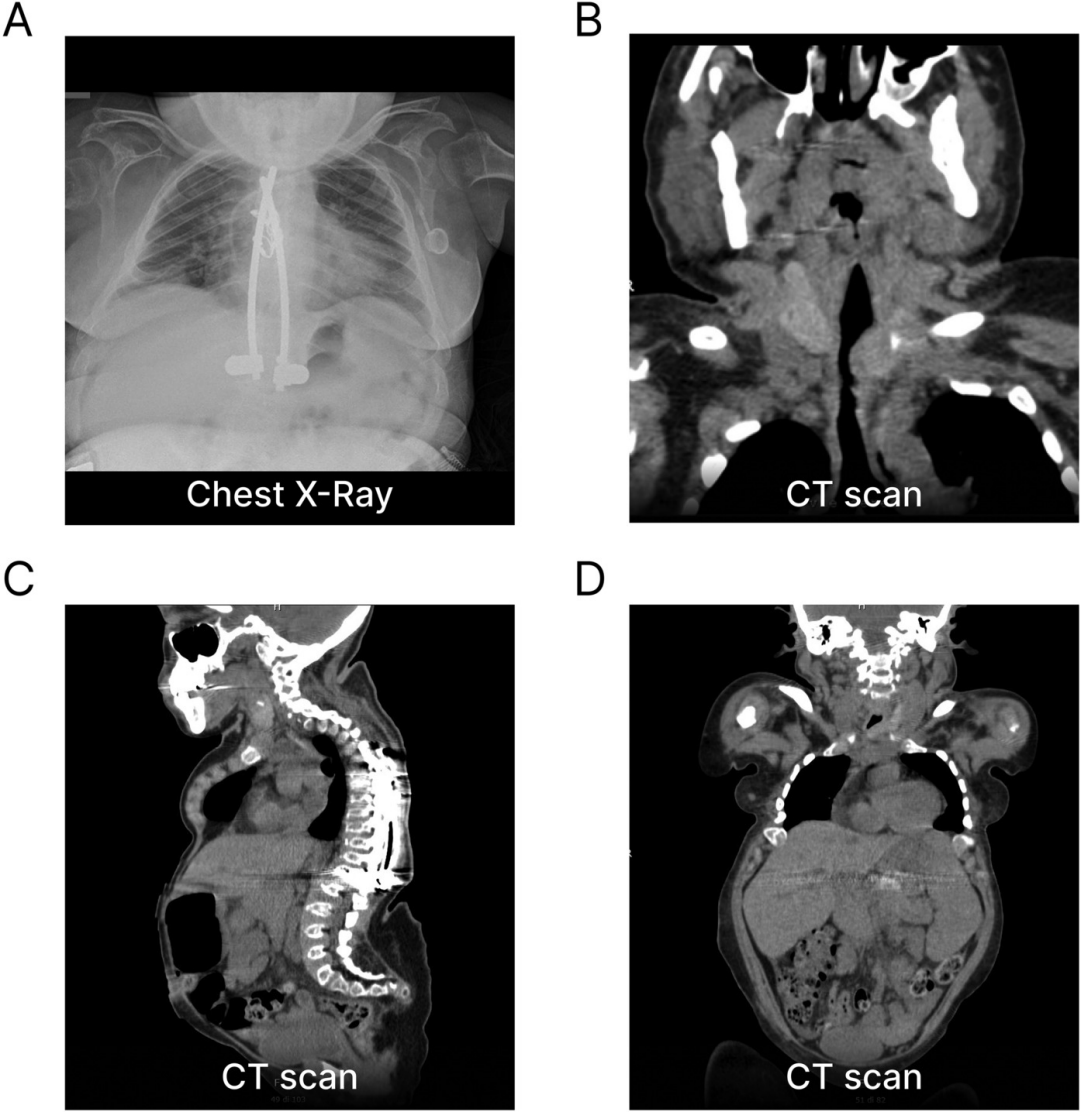
Imaging sections of phenotypic manifestations of our patient with Morquio syndrome. **(A)** Chest x-ray at B-ALL onset showing the absence of a mediastinal mass and evidence of spinal instrumentation with fixation from T9 to L1. **(B)** Frontal CT scan demonstrating a reduced-caliber trachea secondary to extra-luminal GAG accumulation. **(C)** Sagittal CT scan revealing skeletal dysplasia and severe anatomical abnormalities of the vertebral spine and thorax, including marked thoracic kyphoscoliosis and cervical and sacrolumbar hyperlordosis. **(D)** Total-body CT scan at B-ALL onset showing stable hepatosplenomegaly. GAG, glycosaminoglycans.

At a young age, our female patient underwent clinical investigations due to short stature, skeletal dysplasia and gait impairment. She was diagnosed with MPS-IVA at 4.5 years due to reduced lysosomal GALNS activity in leukocytes and increased urinary keratan sulphate excretion. A compound heterozygosity identified in the *GALNS* gene (c.239C > T; p.Ser80Leu and c.850TTC; p.Phe285del) further confirmed the diagnosis. Since the age of 6, the patient presented spastic paraparesis in the lower limbs due to stenosis of the craniocervical junction and of C7-T3 tract, associated with sensory deficits and hypotonia in the upper limbs. She underwent multiple spinal surgeries—including decompressive laminectomy from C1 to T3, fixation of the craniocervical junction and posterior spinal decompression with instrumentation from T9 to L1 (Figures 1A–C)—resulting in partial clinical improvement. The clinical phenotype was of a classic form of MPS-IVA, characterized by low stature (height 100 cm at age 18), bilateral corneal clouding, bilateral transmissive hypoacusia, severe restrictive pneumopathy, hip dysplasia and scoliosis. No heart valve involvement was documented. At the age of 17, she experienced an episode of acute respiratory insufficiency. A chest CT scan at that time revealed a trachea of reduced caliber (Figure 1B) associated with abnormal nocturnal oximetry findings, for which CPAP therapy was prescribed and used when clinically indicated. Due to paraparesis, the patient was highly dependent on carers. She required limited assistance in eating and drinking, self-hygiene, and dressing the upper body part, while required full help to go to the toilet, putting on/taking off clothes from the lower body part and moving around.

At 18 years, an urgent ophthalmologic evaluation was prompted by the acute onset of decreased vision in the right eye and revealed ipsilateral retinal hemorrhages. During the assessment, she reported a one-month history of pallor and asthenia. Blood tests revealed severe anemia, thrombocytopenia and neutropenia, with lymphoblasts in the peripheral blood smears (Hb 52 g/L, platelets 11.0 × 10^9^/L, WBC 3.6 × 10^9^/L, neutrophils 8%, blasts 25%). The patient was thus transferred to our Pediatric Hematology-Oncology Unit with the suspicion of an acute leukemia.

**Figure 2:**
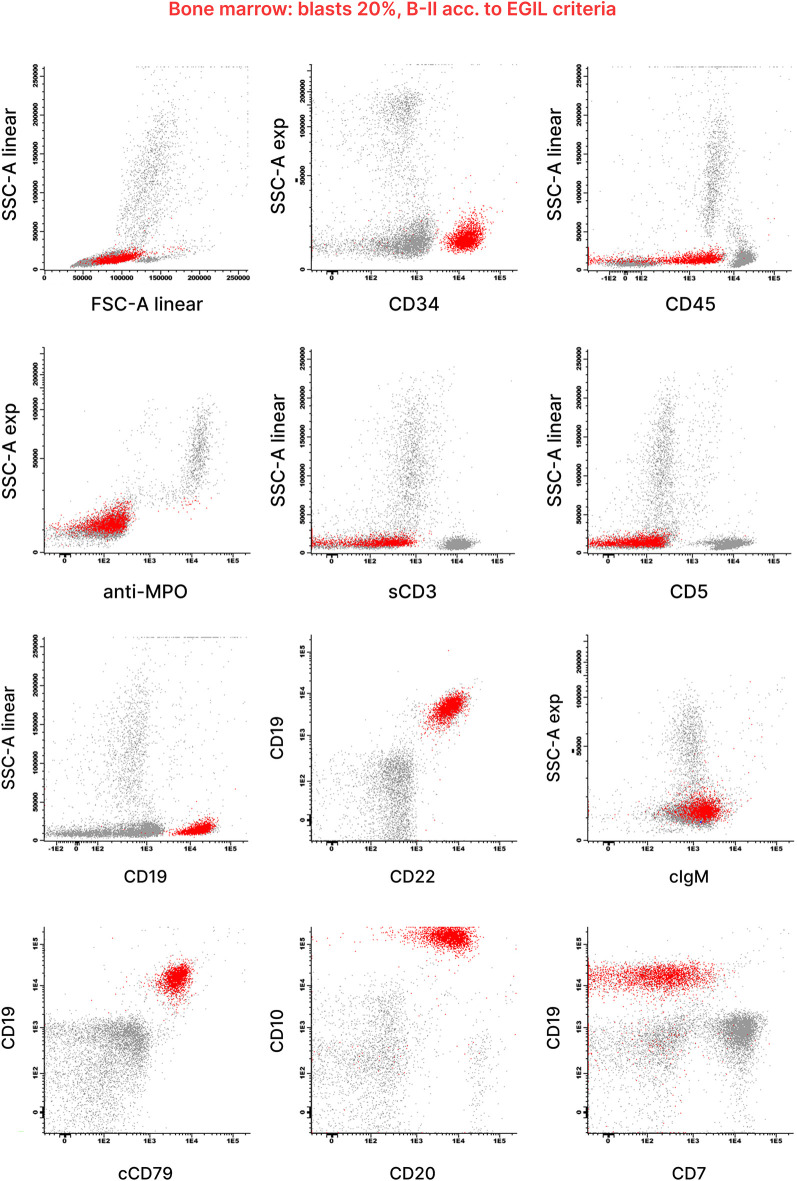
Diagnostic flow cytometry of bone marrow blood from this patient. Flow cytometric analysis of the bone marrow aspirate revealed a blast population (highlighted in red) with uniform expression of B-lymphoid markers CD19, CD20, CD22 and CD10. Antigen expression was analyzed within the blast gate, identified using light scatter parameters and CD45/CD19. The results were generated according to the diagnostic standards approved by the AIEOP-BFM-ALL FLOW-SG, based on the WHO 2008 classification, EGIL criteria and the Bethesda recommendations. Scatter plots were kindly provided by the Hematology Diagnostic Laboratory of Fondazione M. Tettamanti.

At the initial clinical examination, the patient presented with an upper respiratory tract infection and stable hepatosplenomegaly (Figure 1D). Bone marrow (BM) aspiration revealed a monomorphic infiltration of 50% lymphoblasts, confirming the diagnosis of ALL. The immunophenotype was consistent with the B-II subtype (EGIL criteria) (Figure 2). Molecular testing was negative for the translocations t(9;22), t(4;11), t(12;21) and t(1;19). Due to the poor quality of lymphoblast DNA at diagnosis, lymphoid clonality could not be determined for subsequent PCR-based minimal residual disease (MRD) assessment. A chest x-ray ruled out any mediastinal mass and laboratory tests were negative for tumor lysis syndrome. Due to the prior lumbar fixation surgery, lumbar puncture was contraindicated (Figures 1A–C), preventing both the evaluation of CNS involvement and the administration of intrathecal chemotherapy. However, neuroimaging exams were negative for focal leukemic involvement.

**Figure 3:**
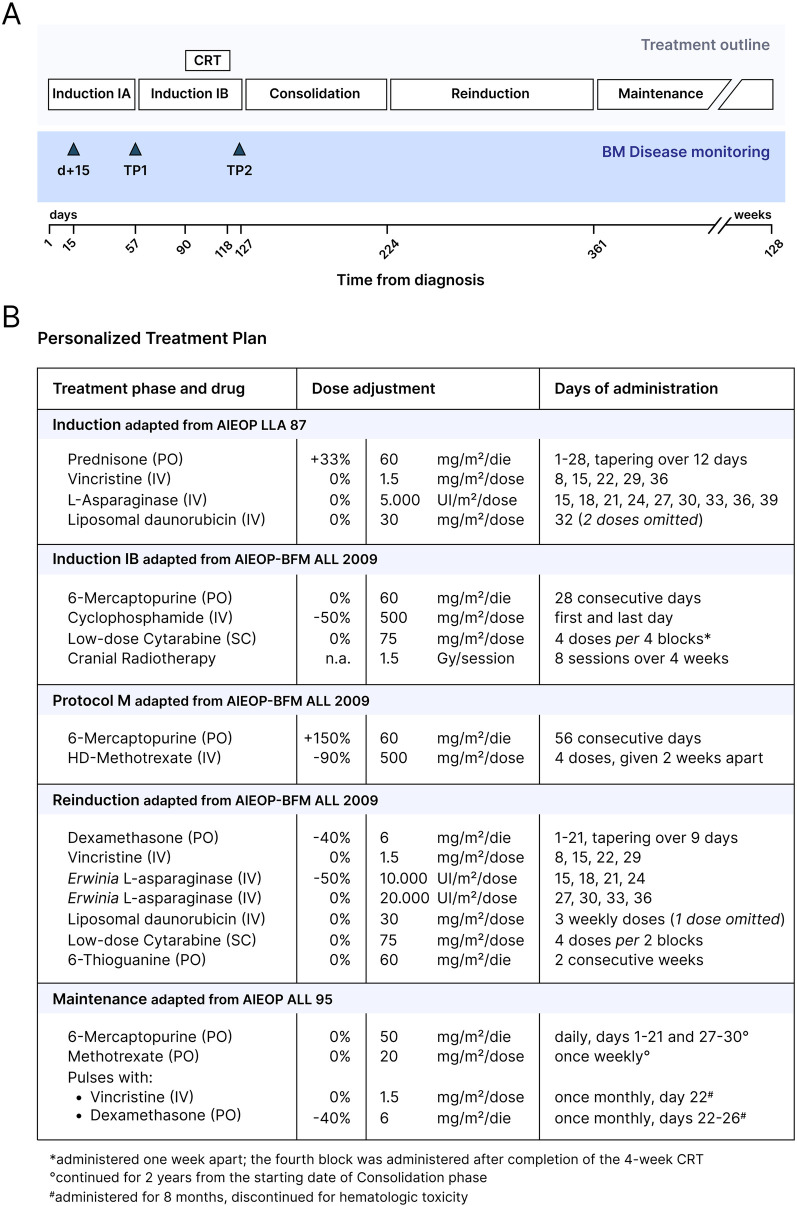
Personalized treatment plan for B-ALL in this patient with Morquio syndrome. **(A)** Overview of the personalized treatment plan and BM disease monitoring designed for the patient. **(B)** Table summarizing the chemotherapy regimen. Dose adjustments with respect to the protocol-based treatment phases are outlined. CRT, cranial radiotherapy; BM, bone marrow; TP1, timepoint 1; TP2, timepoint 2; PO, orally administered; IV, intravenously; SC, subcutaneously.

Considering the severe comorbidities of the patient (reduced body surface area, restrictive pneumopathy, prior lumbar fixation surgery, increased risk of cardio-toxicity), a personalized treatment plan was designed (Figures 3A,B). Induction chemotherapy was based on the AIEOP LLA 87 protocol (NCT00613457) (Figure 3B). A BM aspirate on day +15 showed a MRD of 0.823% by flow cytometry, indicating a good response to the initial treatment. During the ninth dose of native *E. coli* L-Asparaginase, the patient developed a grade 3 hypersensitivity reaction (CTCAE v3.0), requiring early discontinuation of the infusion. On day +32 of Induction, a dose of liposomal daunorubicin (DaunoXome®) was administered under metabolic and echocardiographic monitoring. Due to the development of grade 3 hypertransaminasemia (CTCAE v3.0), further doses of anthracyclines were omitted. The clinical course of this phase was otherwise uneventful. Given the high risk for this patient of developing a potentially life-threatening infection, systemic broad-spectrum antibiotic was administered prophylactically during the initial treatment. A BM aspirate performed at the end of Induction (timepoint 1—TP1) documented the morphological complete remission (CR). Because of the stable clinical conditions and good treatment tolerance, chemotherapy was continued according to the phase IB (Consolidation) of the AIEOP-BFM ALL 2009 protocol (NCT01117441). Due to the inability to perform any intrathecal chemotherapy, CNS prophylaxis was provided via cranial radiotherapy (CRT) upon discontinuation of systemic chemotherapy during this phase (Figure 3A). A BM aspirate performed at timepoint 2 (TP2) confirmed a continuous CR. Therefore, the Intensification phase was personalized on the AIEOP-BFM ALL 2009 protocol M (extracompartimental phase) and consisted of an increased dose of 6-mercaptopurine (60 mg/m^2^/day PO) and four courses of methotrexate (500 mg/m^2^ IV). Reinduction was also derived from the same protocol, and was complicated by an episode of Coronavirus-associated pneumonia, leading to respiratory insufficiency requiring non-invasive ventilatory support. In light of the episode of pneumonia, the two scheduled doses of cyclophosphamide were omitted to avoid myelotoxicity. The intensive phase of chemotherapy was completed approximately one year after diagnosis. Continuation was adapted from the Intermediate-Risk AIEOP ALL 95 protocol (NCT00613457) (Figure 3B). During the early phase of Continuation, the patient developed another episode of pneumonia complicated by acute respiratory insufficiency. Subsequently, considering also several episodes of grade 3 thrombocytopenia (CTCAE v3.0), no further monthly vincristine and five-day dexamethasone pulses were administered. For the view of the whole chemotherapy treatment plan, see Figure 3B. The chemotherapy treatment was discontinued 2.5 years after the diagnosis (Figures 3A,B).

**Figure 4:**
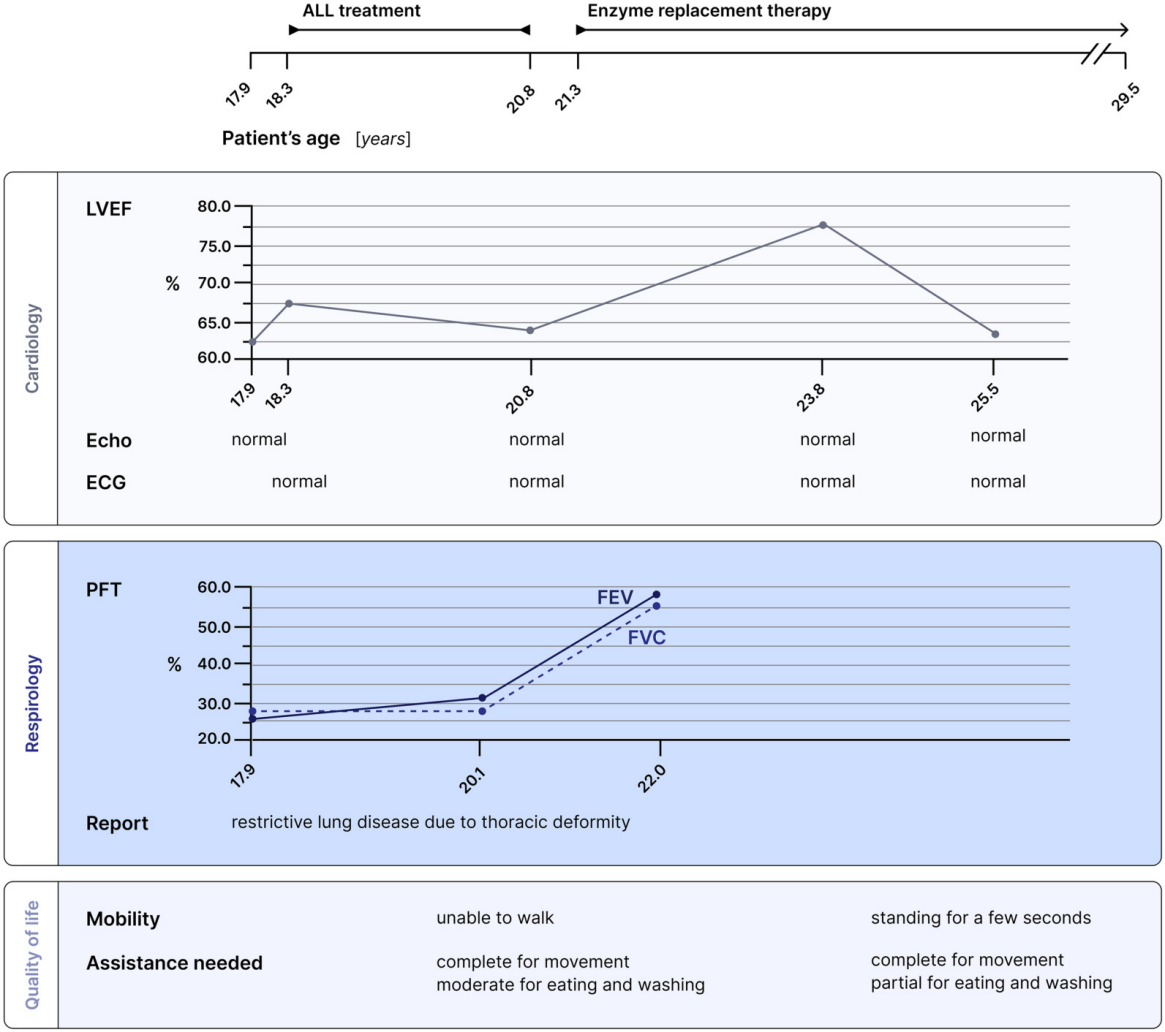
Multidisciplinary follow-up for this patient with Morquio syndrome. Cardiology, respirology and quality of life assessments are presented in relation to both ALL treatment and enzyme replacement therapy. Pulmonary function tests showed improvement, and the patient gained limb strength and the ability to stand for a few seconds under enzymatic therapy. LVEF, left ventricular ejection fraction; ECG, electrocardiogram; PFT, pulmonary function test; FEV, forced expiratory volume; FVC, forced vital capacity.

Upon EMA approval and after the end of chemotherapy, ERT with elosulfase alfa was started based on clinical stability and a joint decision between the patient, her family and physicians. ERT start was followed by an improvement in limb strength and sensitivity, a reduction in secretions of the upper airways, stability in cardiac and pulmonary function, and normalization of urinary GAG (Figure 4).

Currently, the patient remains in continuous CR ten years after the diagnosis of B-ALL. To date, no long term, treatment-related toxicities have been observed (Figure 4).

## Discussion

We report a unique case of successful treatment of B-ALL in a patient with MPS-IVA. The definition of a treatment plan for this patient was particularly challenging due to the rarity of both the underlying metabolic disorder and ALL, the presence of severe comorbidities, the high risk of life-threatening chemotherapy-related complications and the need to deliver effective treatment. To ensure a comprehensive and multidisciplinary approach, the patient was managed at our center, where expertise in both pediatric Hematology-Oncology and Inborn Errors of Metabolism was available. Although our patient had already turned 18 at the time of diagnosis and would normally have been treated in an adult setting, it was agreed to administer a pediatric-oriented and personalized chemotherapy regimen. This decision was supported by encouraging survival outcomes reported over the past 2–3 decades in adolescents and young adults treated with pediatric-inspired regimens compared to adult protocols (11). In addition, our patient had a body surface area typically observed in a 9-year-old girl.

Our patient presented with severe comorbidities, requiring a stepwise approach to defining a personalized treatment plan, which was adjusted on the disease characteristics, the treatment response and the chemotherapy-related toxicities. Indeed, anthracyclines were administered with a reduced schedule and dosage under close metabolic and echocardiographic monitoring; in addition, the liposomal formulation was chosen for its reduced cardiotoxicity (12). To maintain treatment intensity, the native *E. coli* L-asparaginase was substituted with the *Erwinia chrysanthemi* L-asparaginase in Reinduction phase due to an allergic reaction observed at the end of Induction (13). Furthermore, monthly vincristine and dexamethasone pulses—administered during Continuation—were discontinued due to persistent hematological toxicity.

A major concern was the increased risk of pulmonary infection due to the patient's severe restrictive lung disease and thoracic deformity, so that antibacterial prophylaxis with systemic broad-spectrum antibiotics was administered during Induction. Additionally, any episodes of febrile neutropenia were cautiously managed with systemic antibacterial and antifungal therapy.

Another major challenge in this case was the anatomical contraindication to intrathecal chemotherapy due to severe spinal abnormalities and prior lumbar fixation surgery. Considering the high anesthesiologic and infection risks associated with the placement of an intraventricular reservoir, CRT was considered the only viable alternative for CNS prophylaxis. This option was extensively discussed by the treating medical team, given the potential long-term neurocognitive side effects associated with CRT (14), and was finally delivered during phase IB.

Our patient was successfully treated with a personalized, but conventional, chemo-radiotherapy regimen. Nowadays, new effective and less toxic treatment options are available, primarily represented by immunotherapeutic agents, such as Blinatumomab (15–17). The Children's Oncology Group COG AALL1731 clinical trial (NCT03914625) has recently reported that the addition of Blinatumomab to frontline chemotherapy for NCI standard-risk B-ALL with an average or higher risk of relapse significantly improved disease-free survival, leading to the study's early termination at the first interim analysis (17). Notably, also fragile patients, including infants and those with genetic disorders, have been recently reported to particularly benefit from Blinatumomab (15, 16).

To the best of our knowledge, this is the first report of B-ALL in a patient with MPS-IVA. This case emphasizes the importance of individualized treatment planning in patients with ALL and complex comorbidities, for whom a curative-intent approach should rely on multidisciplinary collaboration, tailored therapies, careful monitoring of treatment-related toxicities and response-adapted chemo-radiotherapy.

## Data Availability

The original contributions presented in the study are included in the article/Supplementary Material, further inquiries can be directed to the corresponding author.
